# Coupon Redemption in a National Sample of Individuals Who Use Tobacco and Nicotine Products

**DOI:** 10.1001/jamanetworkopen.2024.29132

**Published:** 2024-08-19

**Authors:** Eugene M. Talbot, Cristine D. Delnevo, Michelle T. Bover Manderski, Kevin R. J. Schroth, Ollie Ganz

**Affiliations:** 1 Rutgers Institute for Nicotine and Tobacco Studies, Rutgers Biomedical and Health Sciences, New Brunswick, New Jersey; 2 Department of Health Behavior, Society and Policy, Rutgers School of Public Health, Piscataway, New Jersey; 3 Department of Biostatistics and Epidemiology, Rutgers School of Public Health, Piscataway, New Jersey

## Abstract

This cross-sectional study investigates coupon redemption in a national sample of US adults who used tobacco and nicotine in the past 30 days.

## Introduction

Coupons are an important marketing strategy for tobacco companies to reduce price barriers for consumers.^1^ The influence of coupon receipt and redemption on tobacco use has been widely studied in those who smoke cigarettes and, more recently, among those who use e-cigarettes. Continued exposure to cigarette coupons encourages smoking progression. It hinders cessation in vulnerable populations already highly targeted by price-manipulating strategies, like women, sexual minority groups, and people of lower socioeconomic status.^2^ A study^3^ using data from the PATH (Population Assessment of Tobacco and Health) Study similarly found these specific groups reporting exposure to coupons for e-cigarette products, regardless of cigarette smoking status. Further exploration of the characteristics of individuals who redeem coupons, particularly for those who use noncigarette tobacco products, is important in the ever-expanding tobacco product marketplace.

## Methods

The Rutgers University Institutional Review Board deemed this cross-sectional study exempt because of minimal participant risk. Participants provided consent before completing the online survey. This study followed the Strengthening the Reporting of Observational Studies in Epidemiology (STROBE) reporting guideline.

Adults who were US residents and aged 18 to 45 years were recruited from a crowdsourcing marketplace, Amazon Mechanical Turk (Amazon), for a cross-sectional web-based survey study in February 2023.^4^ The survey questions can be found in the eAppendix in Supplement 1. We calculated the prevalence of coupon redemption in the previous 30 days among individuals who reported use of various tobacco and nicotine products including cigarettes, cigars, smokeless tobacco (SLT), and nicotine pouches (N = 1146) in the past 30 days. We estimated prevalence ratios (PR) and 95% CIs using a series of modified Poisson regression models,^5^ with CIs excluding 1 indicating statistical significance at *P < *.05.

## Results

This study included 1146 US adults who reported using tobacco in the past 30 days. Among these individuals, 573 (50.0%) were assigned female at birth; 971 were employed (84.8%), 1039 were aged 25 years or older (90.6%), 115 (10%) were non-Hispanic Black individuals, 166 (14.5%) were Hispanic individuals, and 759 were non-Hispanic White individuals (66.2%). Additional overall characteristics of individuals who had used tobacco in the past 30 days and characteristics by coupon redemption status are in Table 1. Self-employment (Model 1: PR, 1.58 [95% CI, 1.18-2.11]; Model 2: PR, 1.69 [95% CI, 1.26-2.26]) or being unemployed (Model 1: PR, 2.08 [95% CI, 1.39-3.11]; Model 2: PR, 2.30 [95% CI, 1.57-3.38]) relative to being employed for wages were positively associated with redeeming a coupon across all models. Participants who had used cigarettes (PR, 2.44 [95% CI, 1.62-3.69]), e-cigarettes (PR, 1.52 [95% CI, 1.18-1.95]), SLT (PR, 1.87 [95% CI, 1.27-2.47]), or nicotine pouches (PR, 1.90 [95% CI, 1.28-2.82]) in the past 30 days were significantly more likely to have also redeemed a coupon for a tobacco product (Table 2). Adjusted PRs increased with a number of products used in the past 30 days in a gradient fashion.

**Table 1.** Distribution of Demographic and Tobacco Use Characteristics Among Participants Who Used Tobacco Products in the Past 30 Days Overall and by Coupon Redemption Status^a^

Characteristic	Participants, No. (%)		
	Total	Redeemed coupons	Did not redeem coupons
Total^b^	1146 (100)	202 (17.6)	942 (82.2)
Age, y			
18-24	107 (9.3)	11 (5.4)	96 (10.2)
25-34	499 (43.5)	85 (42.1)	413 (43.8)
35-45	540 (47.1)	106 (52.5)	433 (46.0)
Sex assigned at birth			
Male	572 (49.9)	86 (42.6)	485 (51.5)
Female	573 (50.0)	116 (57.4)	456 (48.4)
Sexual or gender minority^c^			
Yes	218 (19.0)	56 (27.7)	161 (17.1)
No	928 (81.0)	146 (72.3)	781 (82.9)
Employment status			
Employed for wages	787 (68.7)	117 (57.9)	668 (70.9)
Self-employed	184 (16.1)	52 (25.7)	132 (14.0)
Unemployed	52 (4.5)	18 (8.9)	34 (3.6)
Other	123 (10.7)	15 (7.4)	108 (11.5)
Race or ethnicity			
Hispanic	166 (14.5)	35 (17.3)	130 (13.8)
Non-Hispanic Asian	53 (4.6)	4 (2.0)	48 (5.1)
Non-Hispanic Black	115 (10.0)	12 (5.9)	103 (10.9)
Non-Hispanic White	759 (66.2)	139 (68.8)	620 (65.8)
Non-Hispanic multiracial	40 (3.5)	10 (5.0)	30 (3.2)
Non-Hispanic other^d^	13 (1.1)	2 (1.0)	11 (1.2)
Cigarette smoking status			
Never smoked	170 (14.8)	11 (5.4)	157 (16.7)
Formerly smoked	143 (12.5)	17 (8.4)	126 (13.4)
Smoked some days	453 (39.5)	71 (35.1)	382 (40.6)
Smoked daily	380 (33.2)	103 (51.0)	277 (29.4)
Past 30 d use of product			
Cigarette	903 (78.8)	181 (89.6)	722 (76.6)
E-cigarette	599 (52.3)	126 (62.4)	471 (50.0)
Any cigar	332 (29.0)	79 (39.1)	252 (26.8)
Smokeless	142 (12.4)	57 (28.2)	85 (9.0)
Tobacco free nicotine pouches	86 (7.5)	42 (20.8)	44 (4.7)
Past 30 d polytobacco use, No. of products			
1 (not poly)	564 (49.2)	68 (33.7)	495 (52.5)
2	367 (32.0)	56 (27.7)	310 (32.9)
3	131 (11.4)	32 (15.8)	99 (10.5)
4	49 (4.3)	21 (10.4)	28 (3.0)
5	35 (3.1)	25 (12.4)	10 (1.1)
Trial of VLN cigarettes			
Yes	62 (5.4)	35 (17.3)	27 (2.9)
No	1082 (94.4)	166 (82.2)	914 (97.0)
Trial of tobacco free nicotine pouches			
Yes	144 (12.6)	53 (26.2)	91 (9.7)
No	1002 (87.4)	149 (73.8)	851 (90.3)

Abbreviation: VLN, very low nicotine.

^a^ Coupon redemption was measured with the following questions: (1) In the past 30 days, have you redeemed physical or digital coupons when purchasing tobacco products, including cigarettes, cigars, smokeless tobacco, e-cigarettes, and nicotine pouches? Yes or No; (2) Did you redeem a physical coupon in the past 30 days? Yes or No; (3) Did you redeem a digital coupon in the past 30 days? Yes or No.

^b^ Numbers do not sum to total due to rounding.

^c^ Includes individuals who identify as transgender, or nonstraight or heterosexual in 2 gender and sexuality questions.

^d^ Included individuals who selected other.

**Table 2.** Prevalence and Relative Prevalence of Redeeming a Tobacco Product Coupon in the Past 30 Days Among Participants Who Had Used Tobacco Products in the Past 30 Days^a^

Participants	Prevalence of coupon redemption in the past 30 days, No. (%)	PR (95% CI)	
		Model 1 (N = 1143)	Model 2 (N = 1141)
Age, y			
18-24	11 (10.3)	1 [Reference]	1 [Reference]
25-34	85 (17.1)	1.23 (0.67-2.28)	1.46 (0.82-2.60)
35-45	106 (19.7)	1.36 (0.74-2.50)	1.80 (1.02-3.17)
Sex at birth			
Male	86 (15.1)	1 [Reference]	1 [Reference]
Female	116 (20.3)	1.34 (1.04-1.72)	1.30 (1.01-1.68)
Sexual or gender minority			
Yes	56 (25.8)	1.17 (0.89-1.55)	1.14 (0.86-1.50)
No	146 (15.8)	1 [Reference]	1 [Reference]
Race or ethnicity			
Non-Hispanic Asian	4 (7.7)	0.50 (0.23-1.09)	0.49 (0.22-1.11)
Hispanic	35 (21.2)	0.80 (0.58-1.11)	0.76 (0.55-1.05)
Non-Hispanic Black	12 (10.4)	0.52 (0.31-0.88)	0.51 (0.30-0.86)
Non-Hispanic White	139 (18.3)	1 [Reference]	1 [Reference]
Non-Hispanic other	2 (15.4)	0.97 (0.31-3.07)	0.92 (0.26-3.19)
Non-Hispanic multiracial	10 (25.0)	1.05 (0.62-1.78)	1.11 (0.66-1.86)
Employment			
Employed for wages	117 (14.9)	1 [Reference]	1 [Reference]
Self-employed	52 (28.3)	1.58 (1.18-2.11)	1.69 (1.26-2.26)
Unemployed	18 (34.6)	2.08 (1.39-3.11)	2.30 (1.57-3.38)
Other	15 (12.2)	0.85 (0.52-1.38)	0.91 (0.55-1.48)
Cigarette smoking status			
Never smoked	11 (6.6)	1 [Reference]	NA
Formerly smoked	17 (11.9)	1.82 (0.90-3.69)	NA
Smoked some days	71 (15.7)	1.81 (1.01-3.26)	NA
Smoked daily	103 (27.1)	3.00 (1.68-5.35)	NA
Past 30 d poly use, No. of products			
1, not poly	68 (12.1)	1 [Reference]	NA
2	56 (15.3)	1.27 (0.92-1.74)	NA
3	32 (24.4)	2.11 (1.44-3.09)	NA
4	21 (42.9)	4.05 (2.61-6.27)	NA
5	25 (74.4)	5.20 (3.62-7.47)	NA
Past 30 d use of product			
Cigarettes	181 (20.0)	NA	2.44 (1.62-3.69)
E-cigarettes	126 (21.1)	NA	1.52 (1.18-1.95)
Past 30 d use of product			
Cigars	79 (23.9)	NA	1.08 (0.82-1.44)
SLT	57 (40.1)	NA	1.87 (1.27-2.47)
Tobacco free nicotine pouches	42 (48.8)	NA	1.90 (1.28-2.82)
Trial of VLN cigarettes			
Yes	35 (56.5)	NA	NA
No	166 (15.4)	NA	NA
Trial of tobacco free nicotine pouches			
Yes	53 (36.8)	NA	NA
No	149 (14.9)	NA	NA

Abbreviations: NA, not applicable; PR, prevalence ratio estimated via modified Poisson regression; SLT, smokeless tobacco; VLN, very low nicotine.

^a^ Variance Inflation Factors (VIF) were calculated to assess multicollinearity; all VIFs were less than 4.

## Discussion

To our knowledge, this is the first study to assess the redemption of tobacco product coupons among individuals who use noncigarette tobacco products. While individuals who use cigarettes were indeed more likely to redeem coupons, the same was true, albeit to a lesser degree, for those who used e-cigarettes, SLT, and nicotine pouches. Our findings that women and sexual minority individuals engaged in disproportionately greater coupon redemption align with other studies.^2^

Coupon use increased with the number of products reported and was significantly associated with polytobacco use. Cross-promotion of coupons for different product types may encourage experimentation with new products, and the industry has been shown to engage in crossover direct mail advertising, as recently observed with nicotine pouch products.^6^ Furthermore, our finding that those who were unemployed reported a greater prevalence of redeeming coupons aligns with other research showing that individuals sensitive to prices, such as those of lower socioeconomic status, received more direct mail coupons.^3^ Results should be interpreted in the context of several limitations, including the use of a convenience sample, self-reported data subject to response bias, and the cross-sectional design prohibiting temporal inference. Additionally, we did not ask about what specific product a coupon was redeemed for; however, the use of e-cigarettes, SLT, and pouches was significantly associated with coupon use even after adjustment for cigarette smoking, suggesting that individuals who use tobacco are redeeming coupons of various types and not only cigarettes. We also did not consider whether respondents reside in a state that prohibits tobacco coupon redemption. Thus, additional studies that query specifics about coupons are warranted.

## Notes

Supplement 1. **eAppendix.** Survey Questions

Supplement 2.
Data Sharing Statement
