# Supplementary material for: Coupon Redemption in a National Sample of Individuals Who Use Tobacco and Nicotine Products
Source: JAMA Netw Open. 2024 Aug 19;7(8):e2429132. doi: 10.1001/jamanetworkopen.2024.29132 (PMC11333974; doi:10.1001/jamanetworkopen.2024.29132)
Supplement: Supplement 1. — eAppendix. Survey Questions [file jamanetwopen-e2429132-s001.pdf]

Supplemental Online Content

Talbot EM, Delnevo CD, Bover Manderski MT, Schroth KRJ, Ganz O. Coupon redemption in a national sample of individuals who use tobacco and nicotine product. *JAMA Netw Open*. 7(8):e2429132. doi:10.1001/jamanetworkopen.2024.29132

eAppendix. Survey Questions

This supplemental material has been provided by the authors to give readers additional information about their work.

© 2024 Talbot EM et al. *JAMA Network Open.*

eAppendix. Survey Questions

All Survey Questions Reported

Q2 How old are you? Please type your answer below.

Q4 Have you ever smoked a cigarette, even one or two puffs?
Yes (1)
No (2)

Q5 During the past 30 days, on how many days did you smoke cigarettes? Please type your answer below.

Q8 Do you now smoke cigarettes every day, some days, rarely, or not at all?
Every day (1)
Some days (2)
Rarely (3)
Not at all (4)

Q9 Around this time last year, were you smoking cigarettes every day, some days, rarely, or not at all?
Every day (1)
Some days (2)
Rarely (3)
Not at all (4)

Q24 Have you ever smoked cigarettes fairly regularly?
Yes (1)
No (2)

Q34 A new type of cigarette, VLN, has been authorized to be sold in the U.S. These cigarettes have very low levels of nicotine and are less addictive than regular cigarettes. VLN comes in menthol and non-menthol styles.

Q35 Have you ever tried VLN cigarettes?
Yes (1)
No (2)

Q38 The next questions ask about electronic nicotine vapor products, such as JUUL, Puff Bar, Vuse, and blu. Electronic vapor products include e-cigarettes, vapes, vape pens, e-cigars, ehookahs, hookah pens, and mods. These questions relate to using NICOTINE liquid, pods, or cartridges in electronic vapor products.

Q39 Have you ever used an electronic nicotine vapor product?
Yes (1)
No (2)

Q40 During the past 30 days, on how many days have you used electronic nicotine vapor products? Please type your response below.

© 2024 Talbot EM et al. *JAMA Network Open.*

Q41 Do you currently use electronic nicotine vapor products every day, some days, rarely, or not at all?
Every day (1)
Some days (2)
Rarely (3)
Not at all (4)

Q54 The following questions are about traditional or premium cigars, filtered cigars, and cigarillos. These products go by lots of different names, so please use these descriptions and photos to understand what they are.

Q55 Traditional or premium cigars contain tightly-rolled tobacco that is wrapped in a tobacco leaf. Some common brands of cigars include Macanudo, Romeo y Julieta, and Arturo Fuente, but there are many others.

Q56 Have you ever smoked a traditional or premium cigar, even one time?
Yes (1)
No (2)

Q57 In the past 12 months, have you smoked a traditional or premium cigar?
Yes (1)
No (2)

Q58 On how many of the past 30 days did you smoke a traditional or premium cigar? Please type your response below.

Q59 Do you currently smoke traditional or premium cigars every day, some days, rarely or not at all?
Every day (1)
Some days (2)
Rarely (3)
Not at all (4)

Q69 Cigarillos are medium-sized cigars and sometimes come with plastic or wooden tips. Others have no tips. Common brands of cigarillos include Black & Mild, Swisher Sweets, White Owl, Backwoods and Dutch Masters, but there are many others. The photo below shows examples of cigarillos.

Q70 Have you ever smoked a cigarillo, even one time?
Yes (1)
No (2)

Q71 In the past 12 months, have you smoked a cigarillo?
Yes (1)
No (2)

Q72 On how many of the past 30 days did you smoke a cigarillo? Please type your response below.

Q73 Do you currently smoke cigarillos every day, some days, rarely or not at all?
Every day (1)

© 2024 Talbot EM et al. *JAMA Network Open.*

Some days (2)
Rarely (3)
Not at all (4)

Q81 Filtered cigars are much smaller than cigarillos and have a spongy filter like a cigarette. Cheyenne, Djarum, Prime Time, and Captain Black are common brands of filtered cigars, but there are others.

Q82 Have you ever smoked a filtered cigar, <u>even one time</u>?
Yes (1)
No (2)

Q83 In the past 12 months, have you smoked a filtered cigar?
Yes (1)
No (2)

Q84 On how many of the past 30 days did you smoke a filtered cigar? Please type your response below.

Q92 The next questions are about smokeless tobacco products like chew, snuff, dip, or snus. Common brands include Copenhagen, Grizzly, Skoal, or Camel Snus.

Have you ever used any of the following smokeless tobacco products, even one or two times? Choose all that apply.
Snus pouches (1)
Loose snus, moist snuff, dip, spit, or chewing tobacco (2)
I have never used smokeless tobacco products (3)

Q93 On how many of the past 30 days did you use smokeless tobacco products, like chew, snuff, dip or snus?

Q94 Do you currently use smokeless tobacco products every day, some days, rarely or not at all?
Every day (1)
Some days (2)
Rarely (3)
Not at all (4)

Q101 The next few questions are about a new group of nicotine products – tobacco-free nicotine pouches. Many of these come in pouches and packages that look like snus or other smokeless tobacco, but they are white and do not contain any tobacco in them. They do contain nicotine. Some brands include Zyn, Velo, On!, and Rogue.

Q103 Have you ever tried any of these tobacco-free nicotine pouches?
Yes (1)
No (2)

Q180 On how many of the past 30 days did you use tobacco-free nicotine pouches? Please type your response below.

Q181 Do you currently use tobacco-free nicotine pouches every day, some days, rarely, or not at all?

© 2024 Talbot EM et al. *JAMA Network Open.*

Every day (1)
Some days (2)
Rarely (3)
Not at all (4)

Q350 In the past 30 days, have you redeemed physical or digital coupons when purchasing tobacco products, including cigarettes, cigars, smokeless tobacco, e-cigarettes, and nicotine pouches?
Yes (1)
No (2)

Q630 Did you redeem a physical coupon in the past 30 days?
Yes (1)
No (2)

Q631 Did you redeem a digital coupon in the past 30 days?
Yes (1)
No (2)

Q148 Are you currently…
Employed for wages (1)
Self-employed (2)
Out of work for more than 1 year (3)
Out of work for less than 1 year (4)
A homemaker (5)
A student (6)
Retired (7)
Unable to work (8)

Q178 Are you of Hispanic, Latino, or Spanish origin?
Yes (1)
No (2)

Q179 Which one or more of the following would you say is your race? Select all that apply to you.
White (1)
Black or African American (2)
Asian (3)
Native Hawaiian or other Pacific Islander (4)
American Indian, Alaska Native (5)
Other (specify) (6)

Q629 What is your race or ethnicity? Select all that apply.
White (1)
Hispanic or Latino (4)
Black or African American (5)
Asian (6)
American Indian or Alaska Native (7)
Middle Eastern or North African (8)
Native Hawaiian or Pacific Islander (9)

© 2024 Talbot EM et al. *JAMA Network Open.*

Q149 What sex were you assigned at birth (what the doctor put on your birth certificate)?
Male (1)
Female (2)

Q150 Do you consider yourself to be transgender?
Yes, Transgender, male-to-female (1)
Yes, Transgender, female-to-male (2)
Yes, Transgender, gender non-conforming (3)
No (4)
Don’t know/not sure (5)

Q151 Below is a list of terms that people often use to describe their sexuality or sexual orientation. Please select the term that best applies to you.
Straight/heterosexual (1)
Gay (2)
Lesbian (3)
Bisexual (4)
Queer (5)
Asexual (6)
Pansexual (7)
Questioning/not sure (8)
Another sexual orientation not listed above (please specify) (9)

© 2024 Talbot EM et al. *JAMA Network Open.*
