# Supplementary material for: Coupon Redemption in a National Sample of Individuals Who Use Tobacco and Nicotine Products
Source: JAMA Netw Open. 2024 Aug 19;7(8):e2429132. doi: 10.1001/jamanetworkopen.2024.29132 (PMC11333974; doi:10.1001/jamanetworkopen.2024.29132)
Supplement: Supplement 2. — Data Sharing Statement [file jamanetwopen-e2429132-s002.pdf]

Data Sharing Statement

Data
Data available: Yes
Data types: Deidentified participant data
How to access data: Data are not available in a repository. Since we did not specify in the online consent that the data could be used for secondary data analyses, we are not able to make the data public. A restricted dataset may be requested from Michelle Bover Manderski (ORCID 0000-0003-0000-221X; bovermi@ints.rutgers.edu) and should include a plan for its use.
When available: With publication

Supporting Documents
Document types: None

Additional Information
Who can access the data: Data may be made available to qualified researchers with an approved proposed use after the main findings are published in a peer-reviewed journal.
Types of analyses: Data may be made available to qualified researchers with an approved proposed use.
Mechanisms of data availability: All data sharing will comply with local, state, and federal laws and regulations and may be subject to appropriate human subjects institutional review board approvals.
